# Donor Cell Myeloid Sarcoma

**DOI:** 10.1155/2014/153989

**Published:** 2014-04-14

**Authors:** Mark A. Walshauser, Aileen Go, Payal Sojitra, Girish Venkataraman, Patrick Stiff

**Affiliations:** ^1^Division of Hematology & Oncology, Department of Internal Medicine, Loyola University Medical Center, Maywood, IL 60153, USA; ^2^Division of Hematology & Oncology, Department of Medicine, Cardinal Bernardin Cancer Center, 2160 South First Avenue, Maywood, IL 60153, USA; ^3^Department of Pathology, Loyola University Medical Center, Maywood, IL 60153, USA; ^4^Department of Pathology, The University of Chicago, Chicago, IL 60673, USA

## Abstract

Donor cell derived malignancies are a rare and interesting complication of allogeneic bone marrow transplantation. We present a case of a 56-year-old male with donor cell myeloid sarcoma of the stomach and myocardium.

## 1. Introduction

Treatment of acute leukemia with an allogeneic bone marrow transplant provides a chance for a cure, although relapse is still a cause of mortality posttransplant. Most often when patients relapse, it is from the failure to eradicate their original leukemia. Rarely, patients may develop a leukemia derived from their donor cells, termed donor cell leukemia [1]. We present a unique patient who presented with a donor cell myeloid sarcoma without leukemia in his bone marrow.

## 2. Case

A 56-year-old male was admitted to our hospital 704 days after a human leukocyte antigen- (HLA-) matched sibling donor (sister) allogeneic bone marrow transplant for myelodysplastic syndrome (MDS) with new abdominal pain, weight loss, and night sweats. His myelodysplastic syndrome was classified as refractory anemia with excess blasts-1. He had complex cytogenetics (45,XY,der(5)t(5; 7)(q31; q22), add (11)(p15), add (12)(p13), del (17)(p11.2),−22[17]/46,XY[3]) and positive fluorescence in situ hybridization (FISH) for 5q, 7q, and 11q23. Prior to this hospitalization, he was in complete remission with normal peripheral blood counts, normal trilineage hematopoiesis on bone marrow exam, and complete donor chimerism (>98%) of his peripheral blood.

**Figure 1:**
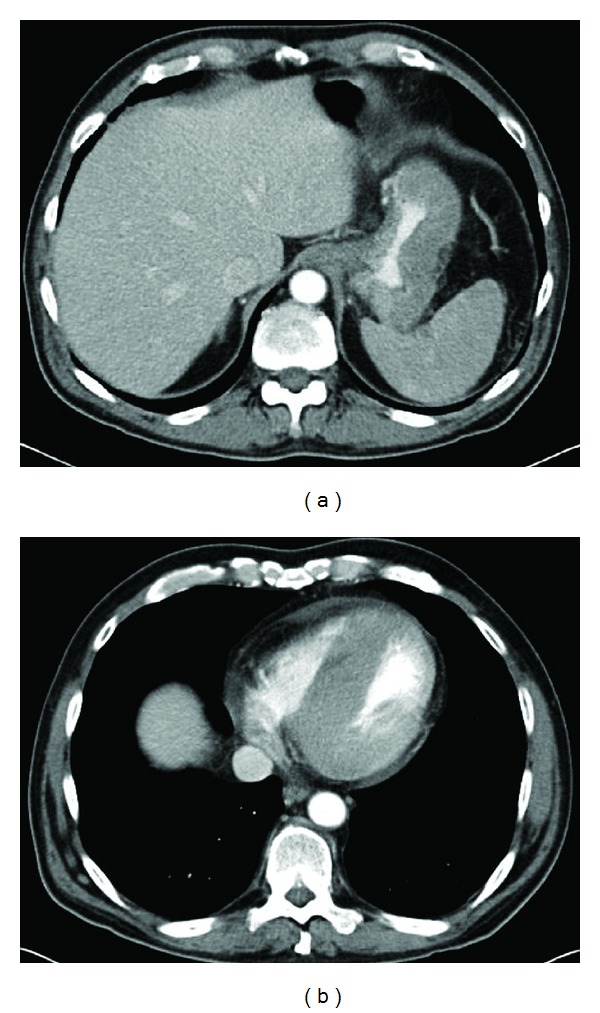
Computed tomography showing diffuse thickening of the gastric wall (a) and interventricular septum (b) due to infiltration of myeloid sarcoma.

**Figure 2:**
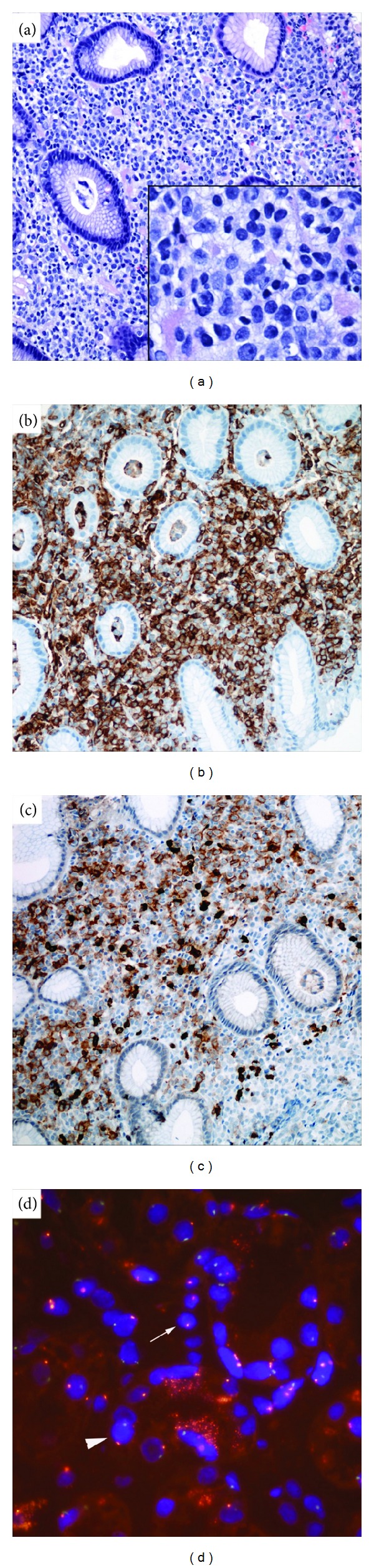
(a) Hematoxylin and eosin stain, showing the gastric biopsy with leukemic infiltrate in the stroma consisting of monomorphous medium sized cells with dispersed but clumped chromatin insinuating in between the normal gastric glands without destruction. Inset shows the high power view (40x magnification) of these leukemic cells. These neoplastic cells express CD34 (b) and CD117 (c). FISH evaluation (d) was performed on this gastric biopsy using X and Y DNA probe set and revealed an XX signal configuration (arrow) in the cells of the leukemic infiltrate which is consistent with female donor chromosome complement and the expected XY configuration (arrowhead) in the gastric tissue consistent with male chromosome complement of this patient.

Computed-tomography scan of the chest and abdomen was performed and revealed gastric wall thickening (Figure 1(a)), retroperitoneal lymphadenopathy and an infiltrating soft tissue mass of left ventricular wall and interventricular septum of the heart (Figure 1(b)). He underwent an esophagogastroduodenoscopy which demonstrated a large ulcerated gastric mass along the greater curvature of the stomach. Biopsy of the mass revealed a leukemic-type infiltrate of monomorphous medium sized cells with dispersed but clumped chromatin insinuating between the gastric glands without destruction of the glands (Figure 2(a)). The neoplastic cells stained positive for CD34 (Figure 1(b)) and CD117 (Figure 2(c)) and negative for CD45, CD79a, and pankeratin consistent with a myeloid sarcoma. A bone marrow aspiration and biopsy at that time showed no leukemia or MDS and a peripheral blood chimerism was greater than 98% donor. FISH evaluation of the gastric mass (Figure 2(d)) using X and Y DNA probe set revealed an XX signal configuration (arrow) in the cells of the leukemic infiltrate consistent with female (donor) cells while the expected XY configuration (arrowhead) in the gastric tissue confirmed male chromosome complement of this patient.

The patient received induction chemotherapy with “7 + 3” (cytarabine 100 mg/m^2^ per day on days 1–7 and daunorubicin 90 mg/m^2^ per day on days 1–3). Fourteen days after induction of chemotherapy, a positron emission tomography scan was done to evaluate the response to chemotherapy. The gastric wall had residual disease with a standard uptake value of 6.3 and the interventricular septum of the heart had increased uptake when compared to the rest of the myocardium. Subsequently, he was reinduced with HiDAC (high dose cytarabine 2000 mg/m^2^ twice a day for 3 days), but he developed neurotoxicity and then bone marrow relapse. He died 12 weeks later. At this time, the donor had a normal CBC.

## 3. Discussion

Donor cell leukemia (DCL) is a rare complication of allogeneic transplantation. The first case was reported in* Lancet* in 1971 by Fialkow et al. [2] and a review of DCL by Wiseman published in 2011 found that only 51 cases of DCL and 13 cases of donor cell MDS have been reported at that time with equal sex distribution [1]. Of the 64 cases, donor grafts originate from a sibling in 74% of cases, matched unrelated donor in 14%, relative other than sibling in 6%, and cord blood in 6% of cases. Acute myelogenous leukemia (AML) is the most common phenotype of DCL reported. Other types of donor cell neoplasms have been reported including, multiple myeloma [3], gingival squamous cell carcinoma [4], and B-cell immunoblastic sarcoma [5]. Donor-derived granulocytic sarcoma has been reported after stem cell transplant very rarely. One reported case of a 35-year-old man was transplanted with HLA full matched sibling (sister) donor for normal karyotype AML [6]. Then, 57 months after his transplant the patient developed a granulocytic sarcoma of the duodenum while a bone marrow biopsy and aspiration showed normal hematopoiesis with full donor chirmerism. Biopsy analysis by short tandem repeats (STR) of the granulocytic sarcoma revealed that 41.2% of the cells where donor in origin. Diagnosing donor-derived granulocytic sarcoma by SRT should be made with caution. It is possible that a reactive neutrophilic or lymphocytic infiltrate of nonleukemic donor-derived cells in the background of host-derived granulocytic sarcoma makes up the 41.2% of abnormal cells seen on the biopsy and therefore would represent host relapse of disease.

DCL is also very rarely reported in recipients of solid organ transplants. In 1999, Bodó et al. reported a patient with donor-derived acute promyelocytic leukemia after a liver transplant [7]. Subsequently, another report noted evolution of an IgA myeloma in a patient 7 years after a renal transplant [8].

The etiology of DCL still remains poorly understood. The most simplistic explanation is that the graft harbored an occult malignancy that is below the threshold of current screening at time of donation and over time develops into overt leukemia. This notion seems tenable in patients who develop donor cell leukemia and their donor develops a malignancy with the same phenotype [3, 9]. However, for many patients who develop DCL, their donors never develop leukemia which raises the possibility that the posttransplant immunologic milieu may be conducive to the growth and evolution of a clonal leukemic process. Viral oncogenesis is implicated in many tumors including posttransplant lymphoproliferative disorders. It is theorized that a virus infects the graft and causes alteration in the genetic machinery, possibly by oncogene integration [1]. In addition, as immunosuppression limits the host tumor surveillance by the immune system, normally appearing neoplastic subclones that would routinely be eliminated by immunosurveillance may develop. Chemotherapy and radiation have long been known to cause secondary malignancies after their use, with the classical example being the development of therapy-related myeloid neoplasms in the setting of alkylators and topoisomerase-II inhibitors. Hematopoietic stem cells are given after chemotherapy during a transplant to minimize this adverse event of chemotherapy, yet it is possible that there may be residual effects of the chemotherapy as patients with DCL have been found to have similar cytogenetic profile in patients with therapy-related leukemia [1].

Establishing the diagnosis of DCL has evolved significantly over the past 40 years paralleling the more frequent use of molecular techniques. Currently used methods of distinguishing between donor and recipient cells include XY FISH (in sex-mismatched donors) and variable number of tandem repeats (VNTR), as well as STR [10]. In our patient, peripheral blood and bone marrow donor chimerism were verified using STR. For his myeloid sarcoma, however, XY-FISH was used to allow direct visualization of which cells are donor and host in origin. Since the mid-1990's, donor chimerism has been detected by either VNTR or STR. In Wiseman's review, 39 of 51 cases of DCL have been diagnosed by one of these two methods. There is limitation to these two methods. Our lab reports that a patient is greater than 98 percent donor chimerism. This is due to the fact that the sensitivity of this test is to detect a population of cells down to 1-2% of the total population. Any population of cells less than 1% of the total population would be missed. Limitations for XY-FISH may occur with gains and loss of sex chromosomes that can be seen in patients with leukemia [11]. In light of such reports, our patient could have lost his Y chromosome and had a gain of X chromosome in his myeloid sarcoma.

The prognosis of DCL has been reported to be similar to patients with other secondary malignancies, and there are no prospective trials investigating outcomes in this patient population. Retrospective reviews of published case reports have found that, similar to primary AML, standard AML therapy in some patients leads to a complete response (CR) and some patients may benefit from a second stem-cell transplantation for long-term control. According to a review of 64 cases of DCL or MDS, 34 cases or 53% of patients died with a median survival of 5.5 months and only 17 or 26% had obtained a CR when the authors had reported their cases [1]. A second transplant has yielded a long-term response in only a single patient [12]. While DCL is associated with a dismal outcome using standard therapy further, efforts at exploring a second reduced intensity transplant are warranted.
